# Construction and validation of a cross-sectional risk classification model for hypoproteinemia in single-center maintenance hemodialysis patient

**DOI:** 10.1038/s41598-025-19913-8

**Published:** 2025-10-15

**Authors:** Kun Zhang, Xiaohan Qu, Shuai Xu, Lu Xu, Xinjian Li, Lei Liu

**Affiliations:** 1 Department of Nephrology, Affiliated Hospital of Jining Medical University, Jining, 272029 Shandong China; 2 Department of Hemopurification, Affiliated Hospital of Jining Medical University, Jining, 272029 Shandong China; 3 Department of General Medicine, Affiliated Hospital of Jining Medical University, 89 Guhuai Road, Rencheng District of Jining, Jining, 272029 Shandong China

**Keywords:** Hemodialysis, Hypoproteinemia, Machine learning, Classification model, SHAP, Medical research, Nephrology

## Abstract

Hypoproteinemia is a common complication across patients receiving maintenance hemodialysis (MHD). Moreover, it is associated with increased risks of cardiovascular events, infection risk, and mortality. This study aimed to construct a classification model for identifying the risk of hypoproteinemia in MHD patients to support precise clinical interventions. To this end, a retrospective analysis was conducted on 288 MHD patients at the Affiliated Hospital of Jining Medical University (January–December 2023). Hypoproteinemia was defined as the primary outcome. Four machine learning (ML) models—Logistic Regression (LR), Random Forest (RF), Support Vector Machine (SVM), and eXtreme Gradient Boosting (XGBoost)—were trained and validated using 3-fold cross-validation. Feature selection was performed using the Least Absolute Shrinkage and Selection Operator (LASSO). Model performance was evaluated using area under the receiver operating characteristic curve (AUC), accuracy, sensitivity, specificity, balanced F-score, and Brier score. SHapley Additive exPlanations (SHAP) was used to interpret the model. The SVM model demonstrated the highest identification performance with an AUC of 0.937. Thirteen key features were identified, with globulin, hemoglobin, β2 microglobulin, prealbumin, and hemodialysis mode being the most significant. In conclusion, the classification model based on ML can accurately identify the risk of hypoproteinemia in MHD patients and provide targeted guidance for early clinical intervention. The combination of SVM and SHAP not only enhances model interpretability but also establishes a scientific foundation for personalized risk classification and the development of precision diagnosis and treatment strategies by visualizing the dynamic effects of key features.

**Supplementary Information:**

The online version contains supplementary material available at 10.1038/s41598-025-19913-8.

## Introduction

End-stage renal disease (ESRD) has become a major global public health issue, and hemodialysis (HD) is the preferred treatment option for patients with ESRD^1^. Hypoproteinemia, a common complication of maintenance hemodialysis (MHD)^2^is defined by plasma total protein levels below 60 g/L or albumin levels below 35 g/L. This condition is a significant risk factor for cardiovascular and cerebrovascular diseases, infections, hospital readmission, and even mortality in patients^3,4^. In recent years, the number of MHD patients in China has increased significantly, with diabetes and aging being the primary driving factors^5^. Chinese patients with MHD face unique challenges. Since hypoproteinemia is associated with a variety of adverse clinical outcomes, early identification of the high-risk population for hypoproteinemia in MHD patients is of great significance for improving prognosis^6^.

At present, most studies have applied traditional methods such as multiple linear regression and logistic regression (LR) to identify the risk of hypoproteinemia. However, it is difficult to manage high-dimensional data and nonlinear relationships^7^. In contrast, machine learning (ML), which combines AI with statistical strengths, has shown significant advantages in predictive models for medicine^8–10^. In the era of medical big data, ML plays a key role in uncovering predictive patterns within complex biological systems^11^. In the field of nephrology, emerging ML models have been used to assess the risk of a variety of diseases^12^. Hu et al.^13^ applied the random forest (RF) model in the single-center MHD cohort and identified age, presence or absence of diabetes, dialysis duration and baseline albumin as key predictors, but this model was not externally validated. By combining with deep learning (DL) optimization algorithm, Yang et al.^14^ developed a multi-factor classification model incorporating hemoglobin, calcium and phosphorus products, but it failed to account for ethnicity-specific factors. Rashid et al.^15^ investigated the impact of malnutrition on the mortality rate of dialysis patients with chronic kidney disease (CKD) through meta-analysis, emphasizing that nutritional status (such as low serum albumin) serves as an independent prognostic predictor and can be used for risk classification. Wang et al.^16^ determined that hemoglobin, red blood cells, serum sodium and serum calcium are independent risk factors for hypoalbuminemia in patients with stage 3–4 CKD, providing reliable, convenient and universally applicable tools for the early identification and intervention of hypoalbuminemia in patients with stage 3–4 CKD. Notably, existing ML studies on hypoproteinemia primarily focus on Western populations, with limited representation of Asian cohorts—particularly Chinese MHD patients, who exhibit unique epidemiological features and distinct lifestyle factors.

Therefore, MHD patients were selected as the research objects in this study. Furthermore, four classical ML algorithms, including LR, RF, support vector machine (SVM), and eXtreme Gradient Boosting (Xgboost), were applied to mine the target dataset and establish a classification model for identifying high-risk hypoproteinemia in MHD patients, based on cross-sectional data. The predictive performance of models under different algorithms was evaluated to identify predictive models with optimal performance. The aim of this study was to identify changes in relevant clinical indicators among patients early in clinical practice, improve the risk perception ability of doctors, and provide scientific bases for precise intervention and treatment of patients in the future.

## Methods

### Study design

Clinical data of 303 maintenance HD patients who visited the Affiliated Hospital of Jining Medical University from January 1, 2023 to December 31, 2023 were collected. This study was approved by the Medical Research Ethics Committee of the Affiliated Hospital of Jining Medical University (Approval No. 2024-02-C005), with all procedures conducted in strict compliance with relevant ethical guidelines and regulatory requirements. Given the non-invasive and anonymous nature of this retrospective study, the requirement for formal informed consent was waived. All human subject data were rigorously anonymized using unique patient identifiers (e.g., study numbers), with direct identifiers such as names, ID numbers, and contact information completely removed to prevent re-identification. This anonymization process adhered to the privacy and data security provisions of the Declaration of Helsinki and the General Data Protection Regulation (GDPR). Data storage and analysis were limited to authorized team members only, ensuring controlled access. All patient data in this study underwent rigorous de-identification to ensure that individual identities remain unidentifiable and traceability to original data is prevented. The dataset was strictly limited to internal team use only and could not be publicly disclosed or shared with external parties. The inclusion criteria of this study were as follows: ① Patients met the diagnostic criteria for ESRD outlined in the International Classification of Diseases^17^; ② Dialysis duration ≥ 3 months; ③ Age ≥ 18 years. The exclusion criteria were as follows: ① Patients with active malignant tumors or other severe diseases; ② Patients with nutritional intake/absorption disorders caused by gastrointestinal disorders, albumin synthesis disorders caused by hepatic decompensation, or albumin loss caused by excessive proteinuria; ③ Patients who developed acute coronary syndrome, trauma, surgery, severe heart failure, or other diseases before routine dialysis; ④ Individuals with incomplete medical records. This study initially included 303 adult patients who received hemodialysis treatment. Based on strict screening criteria, the following patients who did not meet the conditions were ultimately excluded: A total of 15 patients were excluded, including 8 with dialysis duration below 3 months, 2 with combined active malignant tumors, 1 with albumin synthesis disorder caused by liver failure, 3 with severe heart failure before conventional dialysis, and 1 using artificial vascular graft arteriovenous fistula (AVG) as the dialysis access. After screening, a total of 288 subjects who met the research criteria were finally included. The flowchart of patient screening process in models is presented in Figure S1. Given the single-center design and limited follow-up data, we constructed a cross-sectional risk classification tool instead of a longitudinal prediction model. This decision prioritized practicality for immediate clinical implementation while still providing robust risk classification.

## Definition and result

A total of 58 baseline characteristics were collected from each patient for subsequent analysis in this study. These clinical characteristics were reported by clinical clinicians or considered to be closely related to hypoproteinemia. Baseline characteristic information included demographics (sex, age, body mass index (BMI), etc.), dialysis parameters (years of dialysis, type of vascular access, and hemodialysis mode), comorbidity data (diabetes, hypertension), and laboratory indicators (total protein, albumin, hemoglobin, and C-reactive protein (CRP)). A detailed description of all variables is provided in Table S1. All clinical data were collected from the hospital’s medical record system. To minimize bias from sample exclusion, the percentage of missing values for each continuous variable was calculated. For variables with missing values less than 20%, a multiple imputation method was applied for prediction; variables with more than 20% missing values were excluded. The missing and interpolation situations of variables are detailed in attached Figure S2. The primary research outcome of this study was defined as hypoproteinemia, which was determined based on the laboratory test results from the initial data collection node (i.e., the first recorded albumin < 35 g/L or total protein < 60 g/L during the study period).

### Statistical analysis

Patients were grouped into two groups based on the presence or absence of hypoproteinemia. Based on the distribution characteristics, categorical variables were represented as percentages of total variables, while continuous variables were represented as mean ± SD or median (IQR). For categorical variables, the χ² test or Fisher’s exact test was used, while for continuous variables, the t-test or Wilcoxon rank-sum test was applied to compare differences between groups. The Least Absolute Shrinkage and Selection Operator (LASSO) method was employed to screen feature variables related to hypoproteinemia, and significant variables were then used as inputs to train and optimize the models. Four ML algorithms (LR, RF, SVM, and Xgboost) were used to predict the occurrence of hypoproteinemia. To select the optimal classification model for each algorithm with various tuning parameters, 3-fold cross-validation should be applied to the model being optimized. The mean values from 3-fold cross-validation for each algorithm were used to evaluate the receiver operating characteristics (ROC), area under curve (AUC), accuracy, sensitivity, specificity, balanced F-score, and Brier score, so as to evaluate the performance of the ML model. The SHapley Additive exPlanations (SHAP) analysis was performed, and the mean absolute value of the SHAP was calculated to evaluate the importance of each feature. This method employed game theory to evaluate the contribution of each feature. A SHAP chart was visualized to display the key features with the highest importance. Model construction and all statistical analyses were performed using R software (version 4.3.1). A P-value below 0.05 (two-tailed) was deemed as statistically significant.

## Results

A total of 288 patients were enrolled in this study. Their baseline clinical characteristics are shown in Table 1. Baseline characteristics showed that 50 patients (17.4%) experienced hypoproteinemia, while 238 (82.6%) did not. The median age of all patients was approximately 57 years, with the hypoproteinemia group being about 8 years older than the normal albumin group. Compared to patients without hypoproteinemia, a higher proportion of patients with hypoproteinemia applied TCC as their HD access (22.0%) and complicated with coronary heart disease (44.0%). There were significant differences between the two groups in indicators such as prealbumin (*p* < 0.001), globulin (*p* < 0.001), potassium ion (*p* < 0.001), calcium ion (*p* = 0.001), phosphorus ion (*p* = 0.002), hemoglobin (*p* < 0.001), lymphocyte count (*p* = 0.002), and CRP (*p* = 0.002).

**Table 1:** Clinical baseline characteristics, laboratory parameters, and statistical results of maintenance Hemodialysis patients.

	level	Overall	Non-Hypoproteinemia	Hypoproteinemia	*P*
n		288	238	50	
Sex (%)	Male	178 (61.8)	148 (62.2)	30 (60.0)	0.897
	Female	110 (38.2)	90 (37.8)	20 (40.0)	
Age (years)		57.00 [43.75, 66.00]	55.00 [43.00, 66.00]	63.00 [47.25, 69.50]	0.102
BMI (kg/m²)		22.60 [19.84, 25.61]	22.64 [20.00, 25.42]	22.22 [18.69, 26.26]	0.609
Temp (℃)		36.50 [36.30, 36.50]	36.50 [36.30, 36.50]	36.50 [36.40, 36.50]	0.546
HR (per minute)		71.00 [65.00, 78.00]	71.00 [65.00, 78.00]	71.00 [66.25, 80.75]	0.496
SBP (mmHg)		137.59 (22.31)	136.60 (22.61)	142.28 (20.37)	0.102
DBP (mmHg)		80.24 (14.40)	80.11 (14.76)	80.86 (12.69)	0.738
DV (years)		2.00 [1.00, 6.00]	2.00 [1.00, 5.00]	2.50 [0.00, 6.00]	0.459
DF (per week)		3.00 [2.50, 3.00]	3.00 [2.50, 3.00]	3.00 [2.50, 3.00]	0.837
DT (times)		4.00 [4.00, 4.00]	4.00 [4.00, 4.00]	4.00 [3.62, 4.00]	0.115
VA (%)	AVF	255 (88.5)	216 (90.8)	39 (78.0)	0.02
	TCC	33 (11.5)	22 (9.2)	11 (22.0)	
VACT (years)		3.08 [1.49, 6.36]	3.14 [1.65, 6.41]	2.53 [0.58, 6.07]	0.094
HD.Modality (%)	HD	14 (4.9)	12 (5.0)	2 (4.0)	0.036
	HFD	161 (55.9)	141 (59.2)	20 (40.0)	
	HDF	24 (8.3)	20 (8.4)	4 (8.0)	
	HFD + HDF	89 (30.9)	65 (27.3)	24 (48.0)	
HP (%)	No	182 (63.2)	151 (63.4)	31 (62.0)	0.975
	Yes	106 (36.8)	87 (36.6)	19 (38.0)	
ESRD.Cause (%)	Chronic Glomerulonephritis (CGN)	130 (45.1)	105 (44.1)	25 (50.0)	0.504
	Diabetic Nephropathy (DN)	87 (30.2)	71 (29.8)	16 (32.0)	
	Polycystic Kidney Disease (PKD)	16 (5.6)	14 (5.9)	2 (4.0)	
	Hypertensive Nephropathy (HN)	12 (4.2)	12 (5.0)	0 (0.0)	
	Cardiorenal Syndrome (CRS)	11 (3.8)	11 (4.6)	0 (0.0)	
	Lupus Nephritis (LN)	7 (2.4)	5 (2.1)	2 (4.0)	
	Obstructive Kidney Disease (OKD)	5 (1.7)	4 (1.7)	1 (2.0)	
	Tumor-Related Kidney Disease (TRKD)	3 (1.0)	2 (0.8)	1 (2.0)	
	ANCA-Associated Vasculitis (AAV)	2 (0.7)	1 (0.4)	1 (2.0)	
	Other	15 (5.2)	13 (5.5)	2 (4.0)	
DM (%)	No	190 (66.0)	157 (66.0)	33 (66.0)	1
	Yes	98 (34.0)	81 (34.0)	17 (34.0)	
HTN (%)	No	36 (12.5)	25 (10.5)	11 (22.0)	0.046
	Yes	252 (87.5)	213 (89.5)	39 (78.0)	
CAD (%)	No	198 (68.8)	170 (71.4)	28 (56.0)	0.049
	Yes	90 (31.2)	68 (28.6)	22 (44.0)	
CVD (%)	No	217 (75.3)	181 (76.1)	36 (72.0)	0.672
	Yes	71 (24.7)	57 (23.9)	14 (28.0)	
HBV (%)	No	261 (90.6)	214 (89.9)	47 (94.0)	0.526
	Yes	27 (9.4)	24 (10.1)	3 (6.0)	
HCV (%)	No	275 (95.5)	226 (95.0)	49 (98.0)	0.571
	Yes	13 (4.5)	12 (5.0)	1 (2.0)	
CA (%)	No	278 (96.5)	231 (97.1)	47 (94.0)	0.516
	Yes	10 (3.5)	7 (2.9)	3 (6.0)	
Pneum.Inf (%)	No	277 (96.2)	230 (96.6)	47 (94.0)	0.632
	Yes	11 (3.8)	8 (3.4)	3 (6.0)	
CORT (%)	No	268 (93.1)	224 (94.1)	44 (88.0)	0.215
	Yes	20 (6.9)	14 (5.9)	6 (12.0)	
IS (%)	No	270 (93.8)	225 (94.5)	45 (90.0)	0.377
	Yes	18 (6.2)	13 (5.5)	5 (10.0)	
CAKA (%)	No	250 (86.8)	208 (87.4)	42 (84.0)	0.678
	Yes	38 (13.2)	30 (12.6)	8 (16.0)	
ALT (U/L)		7.45 [5.07, 10.43]	7.60 [5.30, 10.80]	6.40 [3.90, 9.67]	0.085
AST (U/L)		10.00 [8.00, 13.00]	10.00 [8.00, 13.00]	11.00 [8.00, 15.00]	0.885
GGT (U/L)		16.00 [11.00, 27.00]	16.00 [11.00, 26.75]	18.50 [12.00, 29.75]	0.241
TBIL (µmol/L)		7.30 [5.80, 9.80]	7.40 [5.82, 9.90]	7.25 [5.28, 8.80]	0.49
PAB (mg/L)		297.52 (71.43)	309.05 (66.30)	242.66 (70.04)	< 0.001
GLO (g/L)		25.00 (4.66)	25.74 (4.05)	21.48 (5.68)	< 0.001
Cr (µmol/L)		945.98 (308.93)	966.83 (287.09)	846.75 (384.95)	0.012
BUN (mmol/L)		23.95 [14.79, 30.00]	24.40 [15.55, 30.17]	21.35 [12.75, 26.10]	0.064
UA (µmol/L)		377.82 (94.82)	382.74 (93.25)	354.40 (99.67)	0.055
β2MG (mg/L)		25.24 [18.41, 32.34]	26.34 [18.72, 32.83]	20.96 [17.94, 27.34]	0.003
ALP (U/L)		93.50 [73.00, 134.50]	95.00 [74.00, 136.00]	80.50 [66.00, 108.75]	0.048
TC (mmol/L)		3.76 [3.22, 4.47]	3.79 [3.26, 4.48]	3.52 [3.10, 4.41]	0.346
TG (mmol/L)		1.15 [0.87, 1.68]	1.15 [0.86, 1.68]	1.08 [0.87, 1.55]	0.432
HDL (mmol/L)		1.12 [0.96, 1.37]	1.14 [0.97, 1.39]	1.06 [0.89, 1.30]	0.078
LDL (mmol/L)		2.21 [1.72, 2.74]	2.24 [1.72, 2.74]	2.21 [1.58, 2.78]	0.724
BG (mmol/L)		4.68 [4.17, 6.30]	4.65 [4.17, 6.02]	4.96 [4.19, 7.27]	0.242
K (mmol/L)		5.00 [4.51, 5.47]	5.06 [4.67, 5.52]	4.60 [3.96, 5.20]	< 0.001
Na (mmol/L)		138.00 [136.00, 140.00]	138.00 [136.00, 140.00]	139.00 [136.00, 141.00]	0.96
Ca (mmol/L)		2.17 [2.04, 2.32]	2.19 [2.06, 2.33]	2.08 [2.01, 2.20]	0.001
P (mmol/L)		1.78 [1.40, 2.26]	1.84 [1.43, 2.28]	1.52 [1.28, 1.87]	0.002
PTH (pg/ml)		340.40 [193.65, 543.78]	351.30 [203.40, 560.32]	281.10 [140.85, 463.28]	0.068
SF (ng/ml)		166.10 [68.94, 324.24]	164.99 [68.37, 318.09]	193.32 [92.04, 401.45]	0.251
Hb (g/L)		111.00 [98.75, 123.00]	115.00 [104.00, 124.75]	95.50 [80.50, 106.75]	< 0.001
RBC (×10^12^/L)		3.67 (0.62)	3.76 (0.56)	3.20 (0.67)	< 0.001
WBC (×10^9^/L)		5.63 [4.57, 7.01]	5.69 [4.70, 7.00]	5.05 [4.21, 7.24]	0.379
PLT (×10^9^/L)		182.00 [131.00, 223.00]	184.50 [132.25, 227.00]	149.00 [114.50, 203.75]	0.017
Neut (×10^9^/L)		3.58 [2.84, 4.66]	3.63 [2.85, 4.62]	3.18 [2.65, 5.39]	0.67
Lym (×10^9^/L)		1.29 [1.02, 1.69]	1.32 [1.05, 1.72]	1.14 [0.83, 1.45]	0.002
Mon (×10^9^/L)		0.37 [0.29, 0.48]	0.37 [0.29, 0.48]	0.38 [0.31, 0.47]	0.376
CRP (mg/L)		3.67 [1.60, 11.00]	3.20 [1.52, 9.11]	7.11 [2.77, 21.08]	0.002

Notes:.

Categorical variables are represented as percentages of total variables, while continuous variables are represented as mean ± SD or median (IQR).

BMI, Body Mass Index; Temp, Body Temperature; HR, Heart Rate; SBP, Systolic Blood Pressure; DBP, Diastolic Blood Pressure; DV, Dialysis Vintage; DF, Dialysis Frequency; DT, Dialysis time; VA, Type of vascular access (AVF, Arteriovenous Fistula; TCC, Tunneled Cuffed Catheters); VACT, Vascular Access Creation Time; HD.Modality, Hemodialysis Modality; HD, Hemodialysis; HFD, High-Flux Hemodialysis; HDF, Hemodiafiltration; HP, Hemoperfusion; ESRD.Cause, Causes of End-Stage Renal Disease (ESRD) (CGN, Chronic Glomerulonephritis; DN, Diabetic Nephropathy; PKD, Polycystic Kidney Disease; HN, Hypertensive Nephropathy; CRS, Cardiorenal Syndrome; LN, Lupus Nephritis; OKD, Obstructive Kidney Disease; TRKD, Tumor-Related Kidney Disease; AAV, ANCA-Associated Vasculitis; Others); HTN, Hypertension; CAD, Coronary Artery Disease; CVD, Cardiovascular Disease; HBV, Hepatitis B Virus; HCV, Hepatitis C Virus; CA, Cancer; Pneum.Inf, Pulmonary Infection; CORT, Corticosteroid; IS, Immunosuppressant; CAKA, Keto Acid Preparation; ALT, Alanine Aminotransferase; AST, Aspartate Transferase; GGT, Gamma-Glutamyl Transpeptidase; TBIL, Total Bilirubin; PAB, Prealbumin; GLO, Globulin; Cr, Creatinine; BUN, Blood Urea Nitrogen; UA, Uric Acid; β2MG, β2 microglobulin; ALP, Alkaline Phosphatase; TC, Total Cholesterol; TG, Triglyceride; HDL, High-Density Lipoprotein; LDL, Low-Density Lipoprotein; BG, Blood Glucose; K, Potassium ion; Na, Sodium ion; Ca, Calcium ion; P, Phosphorus ion; PTH, Parathyroid Hormone; SF, Ferritin; Hb, Hemoglobin; RBC, Red Blood Cell Count; WBC, White Blood Cell Count; PLT, Platelet Count; Neut, Neutrophil Count; Lym, Lymphocyte Count; Mon, Monocyte Count; CRP, C-reactive Protein; TP, Total Protein; ALB, Albumin.

**Fig. 1:**
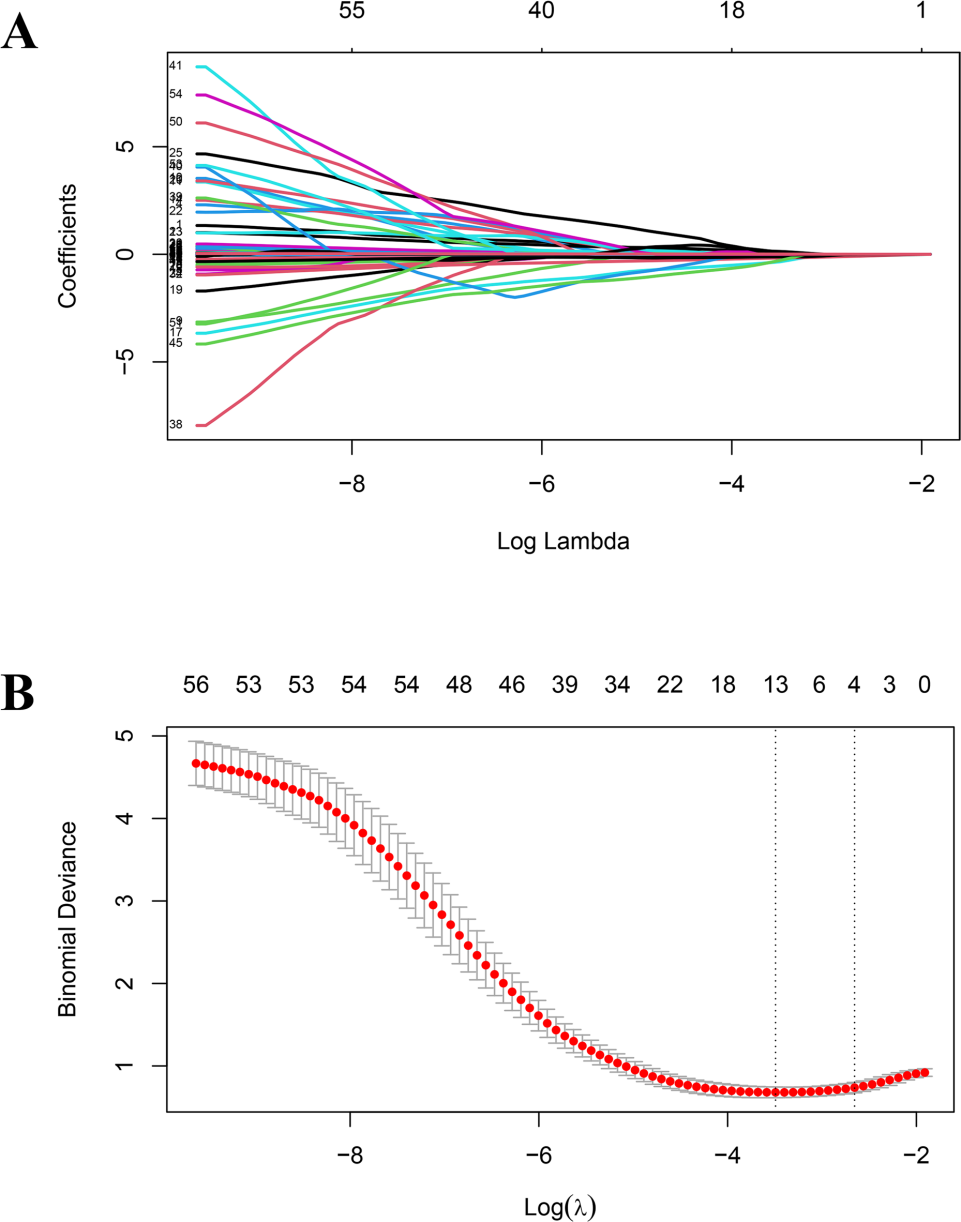
Results of the Least Absolute Shrinkage and Selection Operator (LASSO) method used to filter variables. (**A**) Through 3-fold cross-validation, the coefficients of all classification variables gradually return to zero. (**B**) There are 13 classification variables of non-zero coefficients at the dotted line.

The function selection was performed based on the LASSO method, and the penalty for β coefficient was determined by the tuning parameter λ (λ = 0.03043187). Thirteen feature variables with non-zero coefficients (Fig. 1) were selected, including globulin, hemoglobin, β2 microglobulin, prealbumin, HD mode, potassium, calcium, parathyroid hormone, monocyte count, CRP, gamma-glutamyl transpeptidase, corticosteroid use, and hypertension status. The top 13 features screened by LASSO, along with their regression coefficient values are detailed in Supplementary Table S2.

**Fig. 2:**
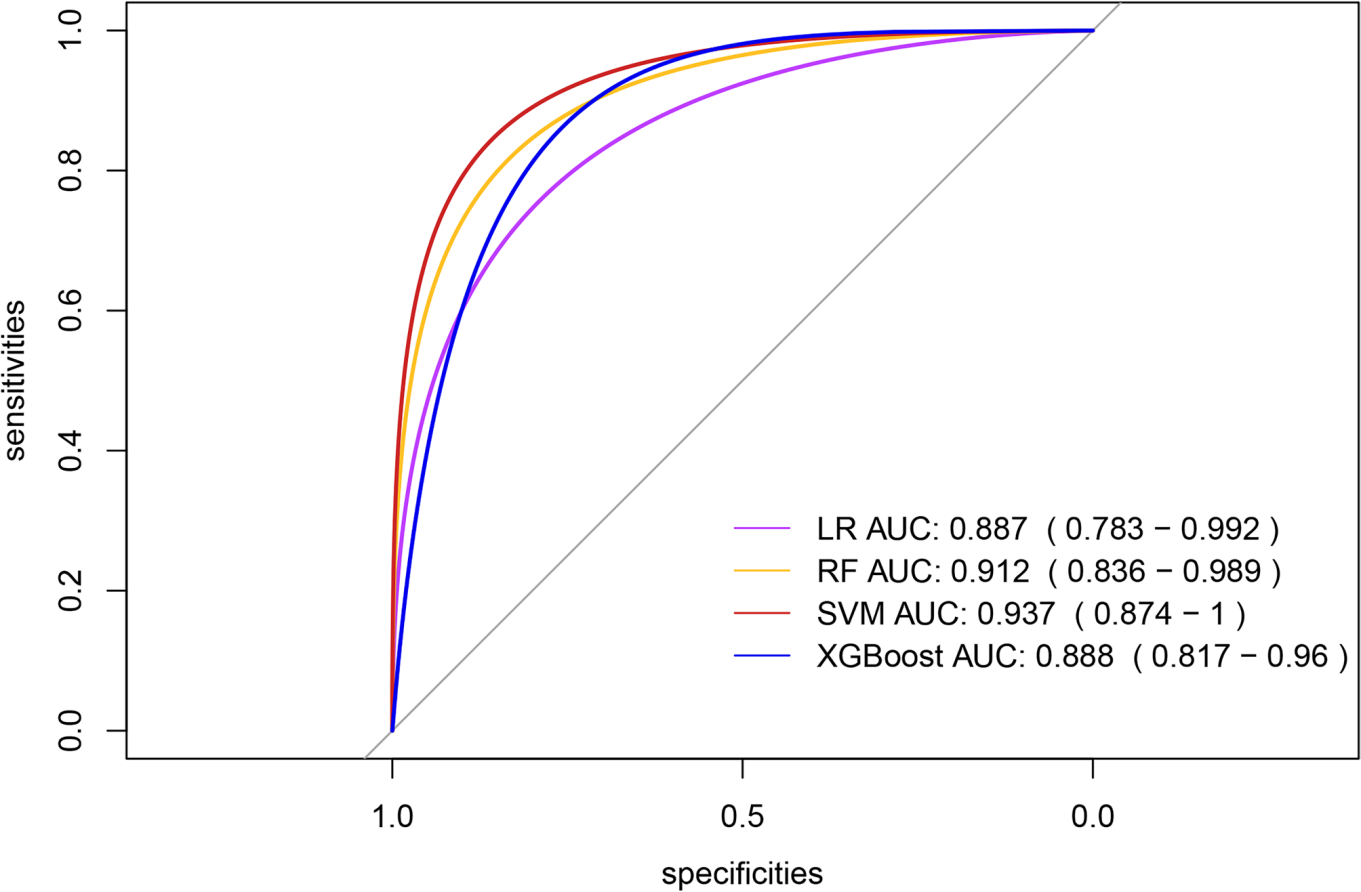
ROC curves for four machine models.

**Fig. 3:**
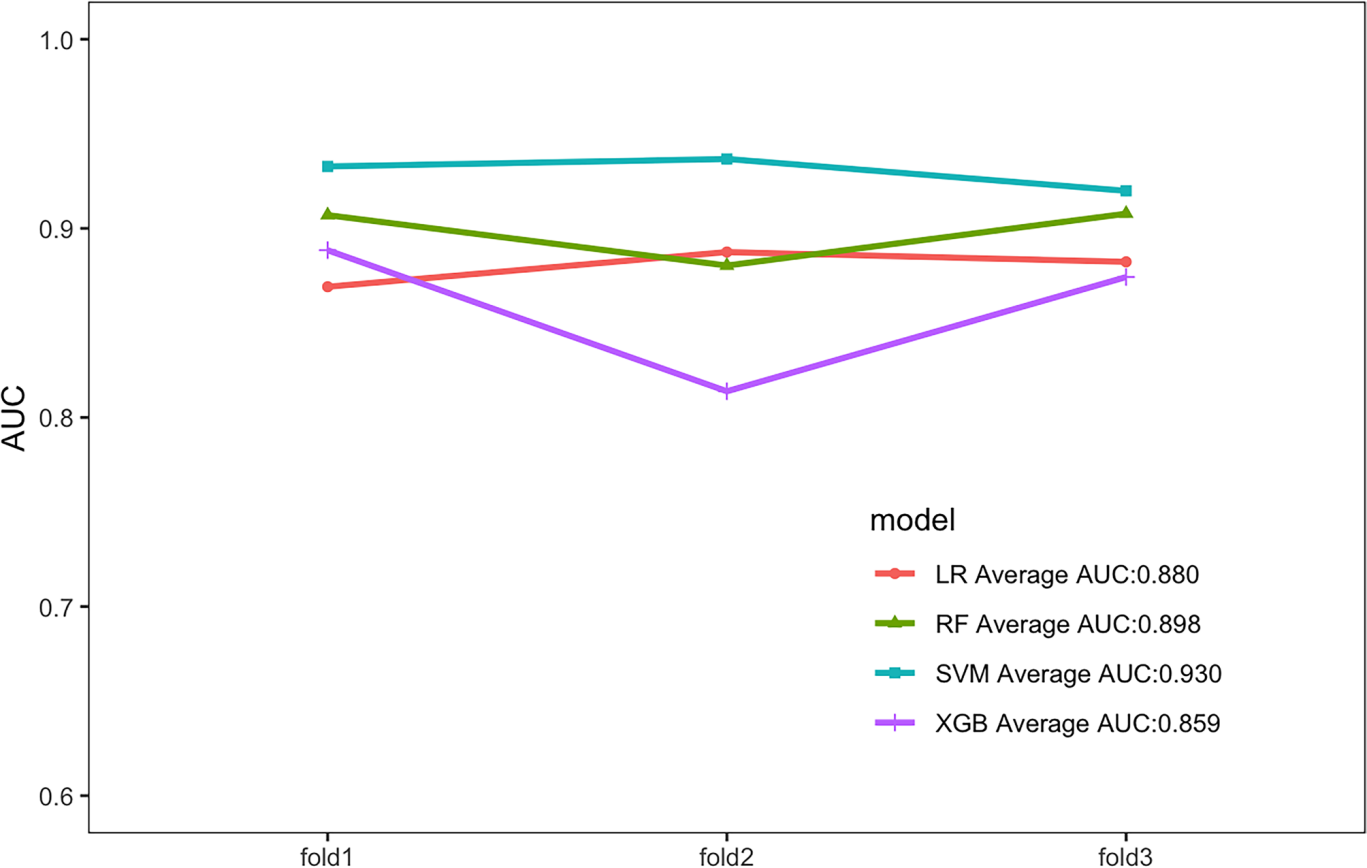
AUC for each fold and mean AUC of four machine models.

**Fig. 4:**
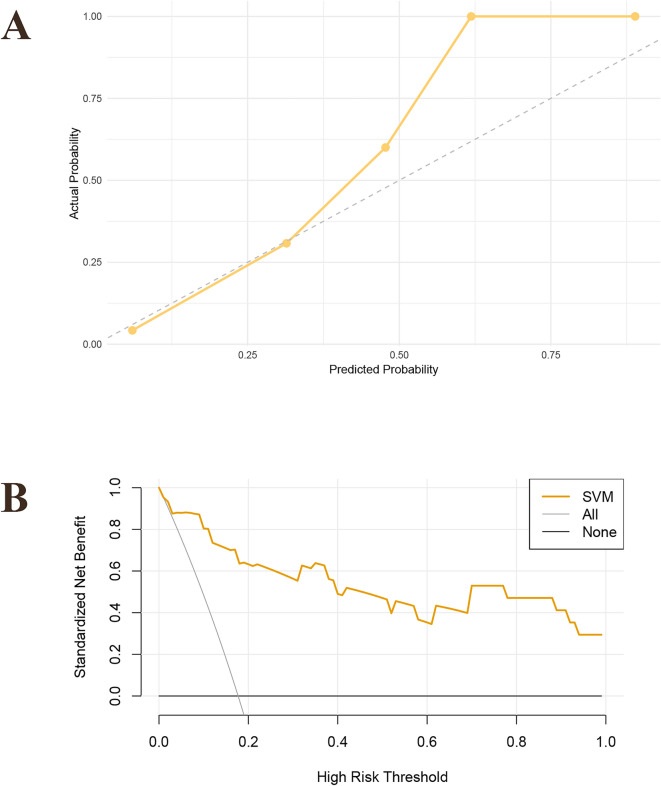
Calibration curve of the SVM model. (**A**) The decision curve analysis of SVM model. (**B**).

Four ML algorithms, namely LR, RF, SVM, and Xgboost, were selected to construct models. The 3-fold cross-validation was applied to the optimized ML models, and the mean value obtained from 3-fold cross-validation for each algorithm was used as the identification result of the algorithm (Table 2). The optimal AUCs of LR, RF, SVM and Xgboost were 0.887, 0.913, 0.937 and 0.888, respectively. It could be found that the AUC values of four models were above 0.85 (SVM > RF > Xgboost > LR), indicating good fitting effect. The mean AUCs of four ML algorithms (LR, RF, SVM, and Xgboost) were 0.880, 0.898, 0.930, and 0.859, respectively. The performance metrics for the four models were summarized as follows: Mean AUC (95%CI): 0.783–0.992, 0.836–0.989, 0.874-1.000, 0.817–0.960; Mean accuracy: 0.861, 0.809, 0.865, 0.750; Mean sensitivity: 0.817, 0.918, 0.898, 0.881; Mean specificity: 0.871, 0.785, 0.857, 0.722; Mean balanced F-scores: 0.670, 0.632, 0.698, 0.554; Mean Brier score: 0.112, 0.095, 0.080, 0.123. ROC curves for 4 machine models were visualized (Fig. 2). After comprehensive evaluation of the four models, it was found that the SVM model demonstrated superior performance across multiple key indicators (particularly in mean AUC, accuracy, F-score, and Brier score), achieving the best fitting effect between predicted and actual results (Fig. 3). The calibration curves showed good consistency among the results, which proved the clinical practicability of the model (Fig. 4A). The decision curve analysis (DCA) indicated that the optimal threshold for defining high-risk hypoproteinemia was 16.5% (Fig. 4B). At this cutoff, the net clinical benefit was maximized, corresponding to a trade-off of approximately five false positives per true positive. In practical terms, this means that the relatively minor cost of unnecessary nutritional counseling or additional laboratory monitoring in a small number of patients is outweighed by the substantial clinical benefit of identifying true high-risk patients who may otherwise progress to severe hypoproteinemia and related complications.

**Table 2:** The 3-fold cross validation average of four machine learning algorithms.

Models	AUC (95%CI)	Threshold	Accuracy	Sensitivity	Specificity	F1-Score	Brier
LR	0.880 (0.783–0.992)	0.169	0.861	0.817	0.871	0.670	0.112
RF	0.898 (0.836–0.989)	0.291	0.809	0.918	0.785	0.632	0.095
SVM	0.930 (0.874-1.000)	0.165	0.865	0.898	0.857	0.698	0.080
XGBoost	0.859 (0.817–0.960)	0.065	0.750	0.881	0.722	0.554	0.123

LR: Logistic Regression; RF: Random Forest; SVM: Support Vector Machine; Xgboost: eXtreme Gradient Boosting.

**Fig. 5:**
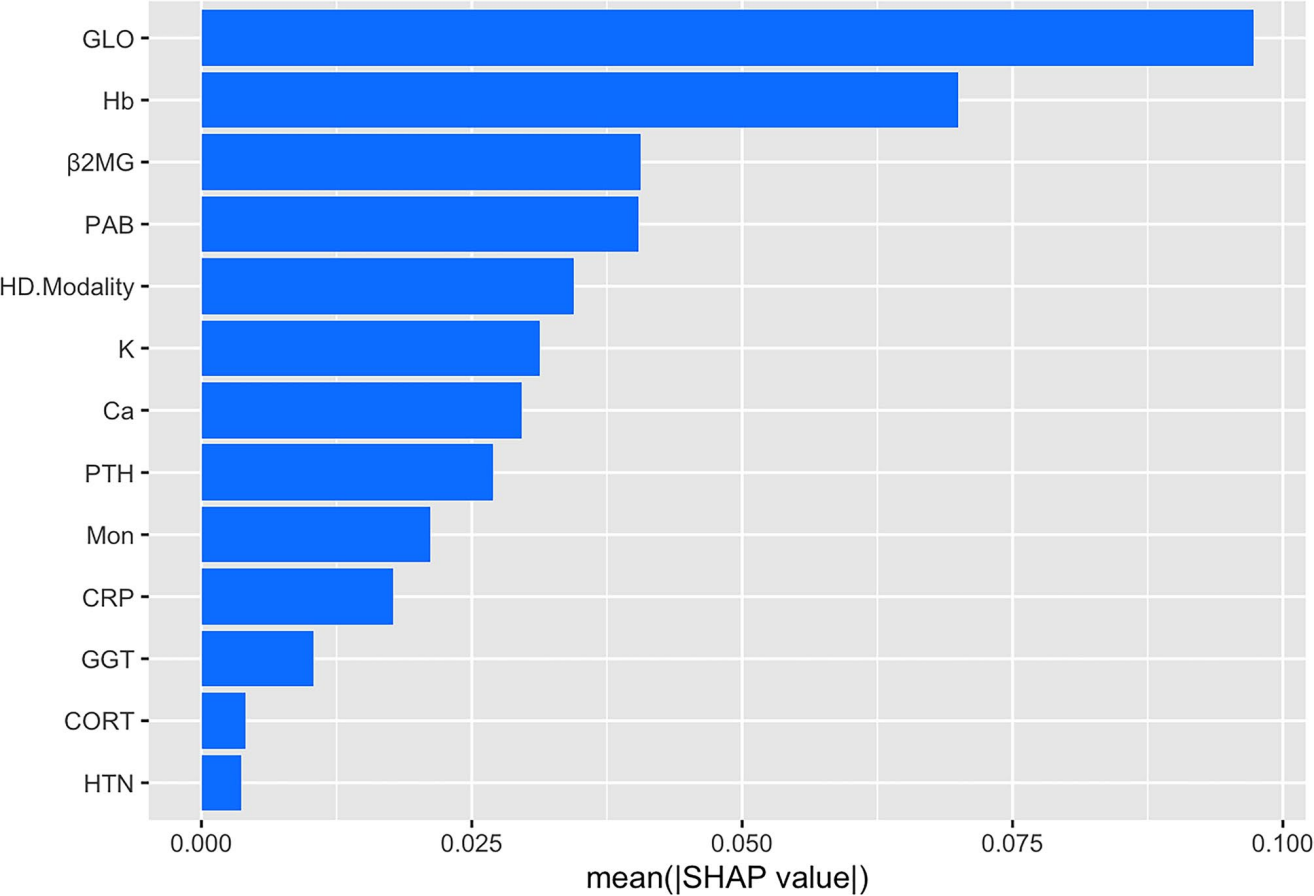
Explanation of the SHAP model: importance ranking of the top 13 variables based on their mean (SHAP value) in the SVM model. The higher SHAP value of a certain feature indicates higher risk of hypoalbuminemia in patients.

Given the variability in feature processing and the differential weighting of identical feature variables across models, SHAP values were applied to assess model interpretability. Given the optimal overall performance of the SVM model, the importance of feature variables was ranked (Fig. 5). The figure identified 13 important feature variables that contribute to hypoproteinemia, including globulin, hemoglobin, β2 microglobulin, prealbumin, HD mode, potassium, calcium, parathyroid hormone, monocyte count, CRP, gamma-glutamyl transpeptidase, corticosteroids use, and hypertension status. The SHAP chart was used to intuitively visualize feature importance in the models and rank the features accordingly. The importance values reflected the extent to which each feature influenced prognosis. The SHAP value indicated that globulin, hemoglobin and prealbumin are the main contributors. The five most critical variables in the SVM model were globulin, hemoglobin, β2 microglobulin, prealbumin, and HD mode.

## Discussion

This study developed four ML classification models using retrospective data to clarify the occurrence and influencing factors of hypoproteinemia in MHD patients. SVM showed the best performance among the four classification models. We have identified the optimal risk threshold for SVM: when the predicted probability ≥ 16.50%, the model achieves the maximum net benefit in distinguishing ‘high-risk’ from ‘non-high-risk’ patients. Based on this threshold, patients were categorized into “high-risk” (predicted probability ≥ 16.50%) and “predicted probability < 16.50%) groups. High-risk patients: It is recommended to prioritize multidisciplinary interventions (e.g., intravenous nutritional support) to reduce the risk of progression to severe malnutrition or complications. Low-risk patients: Routine clinical monitoring (e.g., serum albumin assessment every 3 months) is recommended, with no additional interventions required, thereby optimizing resource allocation. Based on the feature importance of models, the 13 influencing variables screened were high-risk predictors of hypoproteinemia. Among them, prealbumin, globulin, hemoglobin, β2 microglobulin and hemodialysis mode collectively contributed 78% to risk identification. This model accurately classified the risk of hypoproteinemia in MHD patients by real-time identification of high-risk characteristic data.

SHAP values were applied in this study for interpretability analysis of models. The top 5 important factors of features were ranked as follows: globulin, hemoglobin, β2 microglobulin, prealbumin, HD mode. The occurrence of hypoproteinemia in patients with ESRD is closely related to inflammation. Hypoalbuminemia not only impairs erythropoiesis but also contributes to the development of anemia^18–20^. The change trend of hemoglobin in this study was consistent with that of albumin, which further confirmed the correlation between the two indicators. Globulin is mainly composed of immunoglobulin and acute phase protein. The higher level of globulin is correlated to more serious inflammation. Therefore, the level of globulin can be used as an independent prognostic indicator^21,22^. Previous studies have also confirmed a clear association between albumin and globulin^23^. Prealbumin, an important indicator of protein synthesis, exhibits a direct correlation with albumin levels. Early changes in prealbumin concentration can predict subsequent fluctuations in albumin, and its reduction may exacerbate hypoalbuminemia^24–26^. Many studies have confirmed that CRP elevation is closely related to low albumin^27–29^. It is suggested that reducing CRP and improving nutritional status should be the focus of MHD management. Further mechanism analysis reveals that dystrophic-inflammatory syndrome (MICS) is the core pathological mechanism of hypoproteinemia in MHD patients. Patients with hypoproteinemia exhibited significantly decreased prealbumin (*p* < 0.001) and globulin (*p* < 0.001), alongside elevated CRP (*p* = 0.002) and worsened anemia (*p* < 0.001). These findings were highly consistent with the MICS theory proposed by Kalantar-Zadeh et al.^19^. In addition, high-throughput hemodiafiltration was significantly associated with hypoalbuminemia (*p* = 0.036), potentially due to protein loss form the clearance of medium molecular toxins such as β2 microglobulin during high-efficiency dialysis. Previous studies have shown that the albumin leakage of dialysis membranes in this mode can reach 0.8–1.2 g/time^30^. Therefore, the dialysis mode should be selected based on a comprehensive assessment of individual patient needs.

It should be noted that while serum albumin levels < 35 g/L or total protein levels < 60 g/L are commonly used as diagnostic criteria for hypoproteinemia, these two indicators reflect distinct physiological processes. Serum albumin mainly represents the level of hepatic albumin synthesis, while total protein contains albumin and other types of plasma proteins. Although these two indicators are not identical, they have been employed in some clinical studies to assess the risk of hypoproteinemia. For example, in a study on adult patients with congenital heart disease^31^serum albumin levels < 35 g/L and total protein levels < 60 g/L were both used as evaluation indicators for hypoproteinemia, and the two showed a strong correlation. Moreover, a separate on post-stroke patients^32^ has also supported the use of these two parameters as biochemical markers of malnutrition. Based on these findings, we consider it reasonable to adopt serum albumin < 35 g/L and total protein < 60 g/L as diagnostic criteria for hypoproteinemia in this study. To reduce potential confounding effects, we suggest that different laboratories should adjust these thresholds according to their specific patient populations and testing standards, while also considering the specific clinical significance of these two indicators in the local environment, in order to reduce confounding effect during application. Through more detailed analysis and personalized adjustments in different laboratories, the combination of serum albumin and total protein will effectively enhance the early identification of hypoproteinemia.

To control the risk of overfitting, in this study, five-fold cross-validation was adopted to optimize the regularization parameters of LASSO (λ = 0.03043187, finally selected) for variable selection. Bootstrap validation was further incorporated to effectively reduce the risk of model overfitting due to the limited sample size. The 13 modeling variables included in this study are not only readily obtainable in clinical practice but also facilitate the implementation of tailored interventions. Based on these data, we constructed a hypoproteinemia risk classification model for MHD patients, which can realize early warning of hypoproteinemia and help clinicians to adopt timely nutritional support and anti-inflammatory therapy. This facilitates individualized treatment adjustments, ultimately improving patients’ long-term survival rates and quality of life. This model can be integrated into hospital electronic health record (EHR) systems for clinical translation: For example, before each dialysis process, the model can automatically extract feature data (prealbumin, globulin, hemoglobin, etc.) and calculate a risk score with early warning prompts (e.g., “High risk: Nutritional support recommended”), assisting clinicians in rapid decision-making. Additionally, the model can be updated in real-time using dynamic indicators (e.g., albumin changes post-dialysis) to enhance the timeliness of interventions.

The decision curve analysis (DCA) indicated that the optimal threshold for defining high-risk hypoproteinemia was 16.5%. At this cutoff, the net clinical benefit was maximized, corresponding to a trade-off of approximately five false positives per true positive. In practical terms, this means that the relatively minor cost of unnecessary nutritional counseling or additional laboratory monitoring in a small number of patients is outweighed by the substantial clinical benefit of identifying true high-risk patients who may otherwise progress to severe hypoproteinemia and related complications. In addition, it is important to emphasize that this study should be regarded as an exploratory analysis based on a single-center dataset with a relatively small sample size. Although we employed rigorous statistical and machine learning techniques—including LASSO regression, cross-validation, bootstrap resampling, and SHAP interpretability—to mitigate the potential risks of overfitting and selection bias, the findings may still lack full representativeness. Therefore, the results should be interpreted with caution, and future multicenter studies with larger cohorts are warranted to further validate and generalize our conclusions.

It should be pointed out that this study has certain limitations: First, its retrospective, cross-sectional, and single-center design may introduce bias and limit causal inference or generalizability. Although rigorous methods—including LASSO regression, cross-validation, bootstrap resampling, and SHAP interpretability—were applied to mitigate overfitting, the relatively small sample size remains a concern. We adopted the sample size estimation method for clinical prediction models proposed by Riley et al. ^33^. Nevertheless, the available cohort still falls short of the optimal sample size, underscoring the need for multicenter external validation. Second, important unmeasured confounders (e.g., dialysis adherence, home nutritional support, socioeconomic status) were not captured and should be considered in future work. Third, subgroup analyses were exploratory due to limited power and require further confirmation. Despite these limitations, the identified predictors (e.g., prealbumin, globulin, hemoglobin, CRP) are biologically universal, supporting their potential applicability across diverse populations. To strengthen clinical translation, future multicenter prospective studies with larger cohorts are planned to recalibrate and externally validate the model, as well as to iteratively update it with additional variables when available.

## Conclusion

We have constructed classification models that can scientifically quantify the incidence of hypoproteinemia in MHD patients, and have identified 13 characteristic variables closely related to the occurrence of hypoproteinemia. Among these variables, 5 key risk factors, including globulin, hemoglobin, β2 microglobulin, prealbumin, and HD mode, were identified as the most significant contributors to model predictions and the development of hypoproteinemia. This study has verified that SVM based ML models have good performance. The established classification model is helpful for identifying MHD patients with a higher risk of hypoproteinemia, assisting clinicians in providing a theoretical basis for precise diagnosis and treatment of patients, and has important clinical significance for achieving scientific management. Our SHAP-interpretable model demonstrates the potential of EHR-based integration, enabling high-risk MHD patients to undergo early nutritional or inflammatory intervention.

## Supplementary Information

Below is the link to the electronic supplementary material.

Supplementary Material 1

## Data Availability

The original contributions presented in the study are included in the article/Supplementary Materials. Further inquiries can be directed to the corresponding author.
