# Supplementary material for: Construction and validation of a cross-sectional risk classification model for hypoproteinemia in single-center maintenance hemodialysis patient
Source: Sci Rep. 2025 Oct 15;15:36093. doi: 10.1038/s41598-025-19913-8 (PMC12528663; doi:10.1038/s41598-025-19913-8)
Supplement: Supplementary file 1 — Supplementary Material 1 [file 41598_2025_19913_MOESM1_ESM.docx]

**Supplementary materials**

**
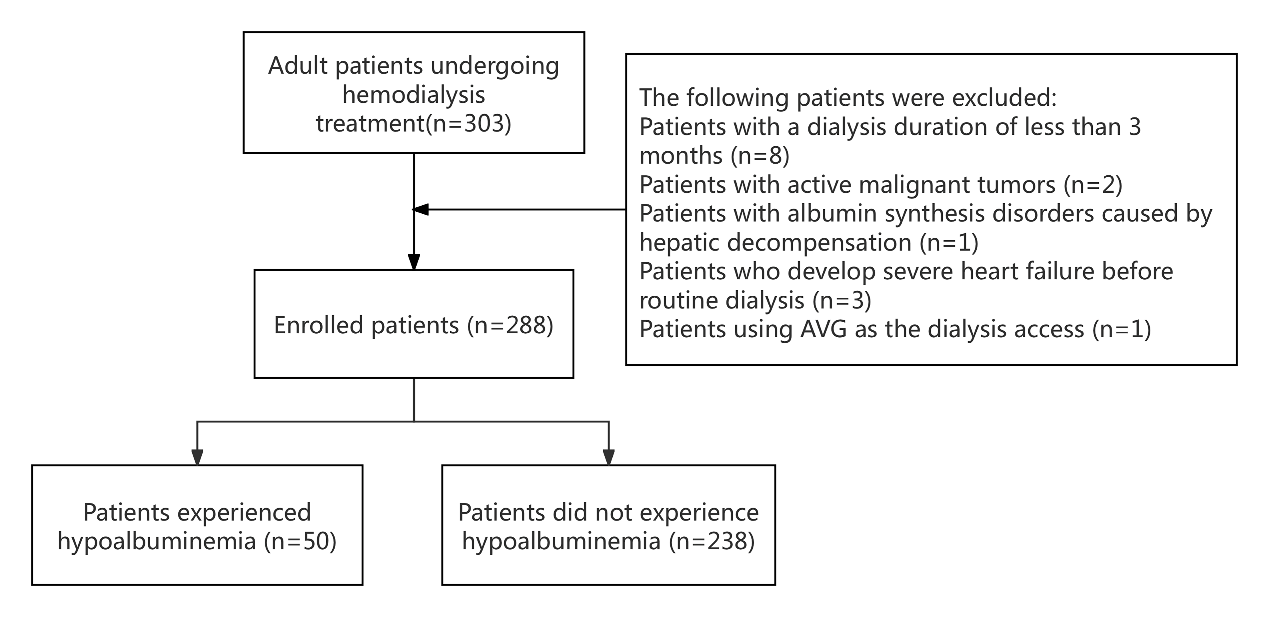
**

**Figure S1** Flowchart of patient screening process.

**
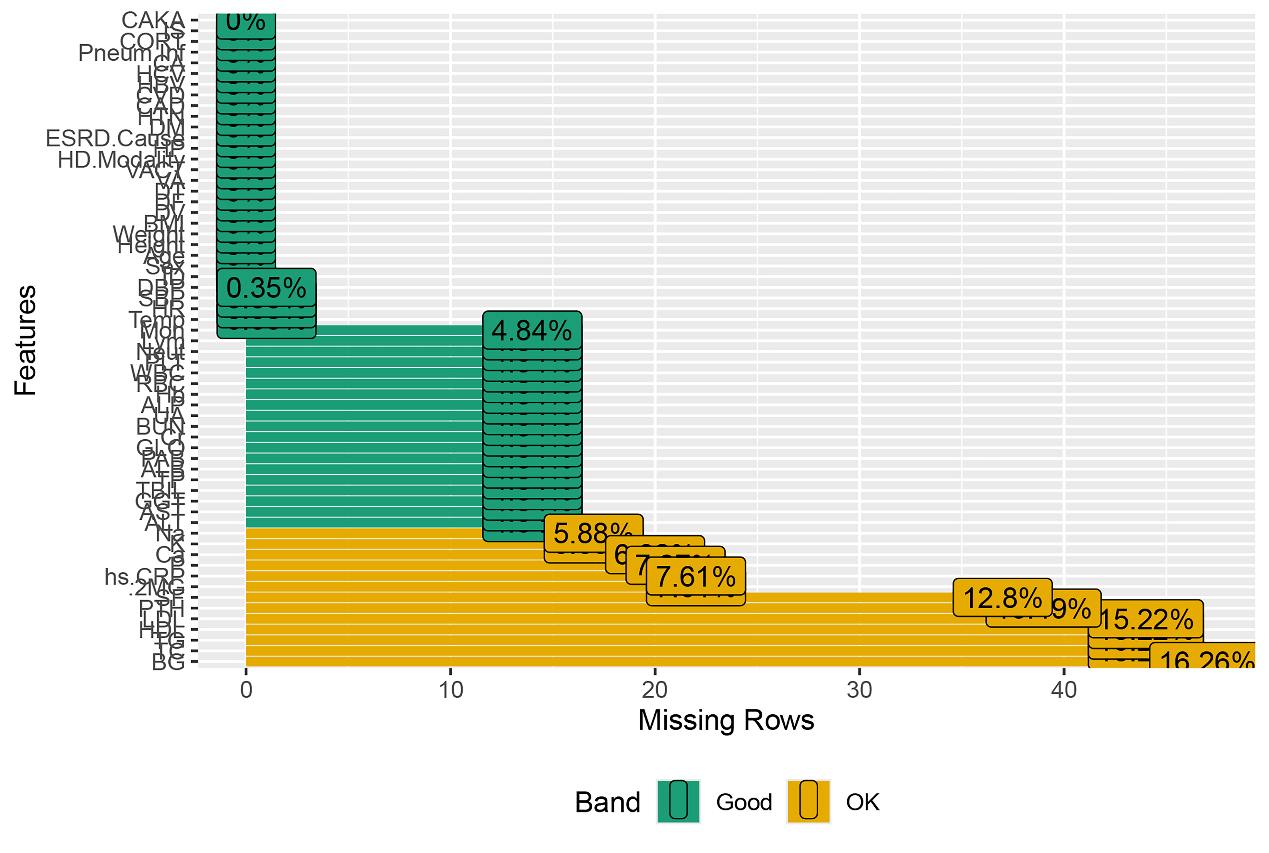
**

**Figure S2** Visualization of missing variables and imputation details.

**Table S1** Included in the list of patients' baseline characteristics

| **Category** | **Variable Name** | **Type** | **Unit/Description** |  |
| --- | --- | --- | --- | --- |
| **Demographics** | Gender | Categorical | Male/Female |  |
|  | Age | Continuous | Years |  |
|  | Body Mass Index (BMI) | Continuous | kg/m² |  |
| **Clinical Characteristics** | Body Temperature | Continuous | ℃ |  |
|  | Heart Rate | Continuous | Beats per minute |  |
|  | Systolic Blood Pressure | Continuous | mmHg |  |
|  | Diastolic Blood Pressure | Continuous | mmHg |  |
| **Dialysis Parameters** | Dialysis Vintage | Continuous | Years |  |
|  | Dialysis Frequency | Continuous | Sessions per week |  |
|  | Dialysis Time per Session | Continuous | Hours |  |
|  | Vascular Access Type | Categorical | AVF/TCC |  |
|  | Vascular Access Creation Time | Continuous | Years |  |
|  | Hemodialysis Modality | Categorical | HD/HFD/HDF/Combined |  |
|  | Hemoperfusion Use | Categorical | Yes/No |  |
| **Comorbidities** | Diabetes Mellitus | Categorical | Yes/No |  |
|  | Hypertension | Categorical | Yes/No |  |
|  | Coronary Artery Disease | Categorical | Yes/No |  |
|  | Cerebrovascular Disease | Categorical | Yes/No |  |
|  | Hepatitis B/C | Categorical | Yes/No |  |
|  | Active Cancer | Categorical | Yes/No |  |
|  | Pulmonary Infection | Categorical | Yes/No |  |
| **Medications** | Corticosteroid Use | Categorical | Yes/No |  |
|  | Immunosuppressant Use | Categorical | Yes/No |  |
|  | Keto Acid Supplementation | Categorical | Yes/No |  |
| **Laboratory Parameters** | Alanine Aminotransferase (ALT) | Continuous | U/L |  |
|  | Aspartate Transferase (AST) | Continuous | U/L |  |
|  | Gamma-Glutamyl Transferase (GGT) | Continuous | U/L |  |
|  | Total Bilirubin (TBIL) | Continuous | µmol/L |  |
|  | Prealbumin (PAB) | Continuous | mg/L |  |
|  | Globulin (GLO) | Continuous | g/L |  |
|  | Serum Creatinine (Cr) | Continuous | µmol/L |  |
|  | Blood Urea Nitrogen (BUN) | Continuous | mmol/L |  |
|  | Uric Acid (UA) | Continuous | µmol/L |  |
|  | β2-Microglobulin (β2MG) | Continuous | mg/L |  |
|  | Alkaline Phosphatase (ALP) | Continuous | U/L |  |
|  | Total Cholesterol (TC) | Continuous | mmol/L |  |
|  | Triglycerides (TG) | Continuous | mmol/L |  |
|  | High-Density Lipoprotein (HDL) | Continuous | mmol/L |  |
|  | Low-Density Lipoprotein (LDL) | Continuous | mmol/L |  |
|  | Blood Glucose (BG) | Continuous | mmol/L |  |
|  | Potassium (K) | Continuous | mmol/L |  |
|  | Sodium (Na) | Continuous | mmol/L |  |
|  | Calcium (Ca) | Continuous | mmol/L |  |
|  | Phosphorus (P) | Continuous | mmol/L |  |
|  | Parathyroid Hormone (PTH) | Continuous | pg/mL |  |
|  | Ferritin (SF) | Continuous | ng/mL |  |
|  | Hemoglobin (Hb) | Continuous | g/L |  |
|  | Red Blood Cell Count (RBC) | Continuous | ×10¹²/L |  |
|  | White Blood Cell Count (WBC) | Continuous | ×10⁹/L |  |
|  | Platelet Count (PLT) | Continuous | ×10⁹/L |  |
|  | Neutrophil Count | Continuous | ×10⁹/L |  |
|  | Lymphocyte Count | Continuous | ×10⁹/L |  |
|  | Monocyte Count | Continuous | ×10⁹/L |  |
|  | C-Reactive Protein (CRP) | Continuous | mg/L |  |
|  | Total Protein (TP) | Continuous | g/L |  |
|  | Albumin (ALB) | Continuous | g/L |  |
| **Notes:** Abbreviations: AVF: Arteriovenous Fistula; TCC: Tunneled Cuffed Catheters; HD: Hemodialysis; HFD: High-Flux Hemodialysis; HDF: Hemodiafiltration. Data Preprocessing: Variables with >20% missing values were excluded; remaining missing values were imputed using multiple imputation (R package mice). Ethical Considerations: All data were anonymized and extracted from electronic health records with institutional ethics approval (No. 2024-02-C005). | | | |  |
|  |  |  |  |  |

**Table S2** Top 13 LASSO-selected features and their regression coefficients.

|  | **s1** |
| --- | --- |
| (Intercept) | 9.312371E+00 |
| Sex | . |
| Age | . |
| BMI | . |
| Temp | . |
| HR | . |
| SBP | . |
| DBP | . |
| DV | . |
| DF | . |
| DT | . |
| VA | . |
| VACT | . |
| HD.Modality | 1.331128E-01 |
| HP | . |
| ESRD.Cause | . |
| DM | . |
| HTN | -3.466884E-01 |
| CAD | . |
| CVD | . |
| HBV | . |
| HCV | . |
| CA | . |
| Pneum.Inf | . |
| CORT | 6.738567E-02 |
| IS | . |
| CAKA | . |
| ALT | . |
| AST | . |
| GGT | 1.234374E-03 |
| TBIL | . |
| PAB | -8.702297E-03 |
| GLO | -1.717016E-01 |
| Cr | . |
| BUN | . |
| UA | . |
| β2MG | -7.378623E-03 |
| ALP | . |
| TC | . |
| TG | . |
| HDL | . |
| LDL | . |
| BG | . |
| K | -1.190894E-01 |
| Na | . |
| Ca | -2.837163E-01 |
| P | . |
| PTH | -6.623633E-05 |
| SF | . |
| Hb | -2.731070E-02 |
| RBC | . |
| WBC | . |
| PLT | . |
| Neut | . |
| Lym | . |
| Mon | 9.136387E-02 |
| CRP | 1.041356E-02 |
